# Comparative Photosynthetic Induction Reveals Stomatal Limitation and Reduced Efficiency in *Digitalis purpurea* Versus *Cucumis sativus*

**DOI:** 10.3390/biology14101445

**Published:** 2025-10-20

**Authors:** Yunmin Wei, Xiaohong Xiang, Wei Jin, Haifeng Xiong, Lihong Tan

**Affiliations:** 1 Key Laboratory of Plant Genetics and Molecular Breeding, Zhoukou Normal University, Zhoukou 466001, China; weiym1024@163.com (Y.W.); jinw877@126.com (W.J.); 17839721223@163.com (H.X.); 2 College of Life Science and Agronomy, Zhoukou Normal University, Zhoukou 466001, China; 3 College of Pharmacy & College of Traditional Chinese Medicine, Chongqing Medical and Pharmaceutical College, Chongqing 401331, China; 20116@cqmpc.edu.cn

**Keywords:** photosynthetic induction, stomatal limitation, biochemical limitation, *Digitalis purpurea*, *Cucumis sativus*

## Abstract

**Simple Summary:**

*Digitalis purpurea*, commonly known as foxglove, is an important medicinal plant that produces heart medications. However, it grows slowly compared to food crops, limiting medicine production. This study investigated why *D. purpurea* has poor growth by comparing it with *Cucumis sativus* plants, a fast-growing crop. We found that when light conditions change from low to high, *D. purpurea* takes three times longer than *Cucumis* to adjust its photosynthesis machinery. Limitation analysis revealed that *D. purpurea* was predominantly limited by stomatal functions, which control gas exchange for photosynthesis, responding too slowly to light changes. Understanding these limitations helps scientists develop better growing methods for *D. purpurea*, potentially increasing medicine production through improved greenhouse lighting strategies or breeding programs targeting faster stomatal responses.

**Abstract:**

*Digitalis purpurea*, valued for its cardiotonic glycosides, remains an important medicinal species. Optimizing cultivation to enhance photosynthetic efficiency is critical for improving both biomass accumulation and metabolite yield. In this study, we compared the photosynthetic induction responses of *D. purpurea* from low light to high light with those of *Cucumis sativus*, a high-performance reference species, to identify key physiological constraints. Compared with *Cucumis*, *D. purpurea* exhibited lower net photosynthetic rate (*A*) and maximum carboxylation rates (*V_cmax_*) under both induction and steady-state conditions (*A*_f_ and *V_cmax_*_f_). The time required to reach steady-state photosynthesis was substantially longer in *D. purpurea*, resulting in significantly lower cumulative carbon gain (20.6 vs. 28.8 mmol m^−2^) and a higher carbon loss ratio (10.7% vs. 6.8%). In addition, the averaged WUE_i_ during induction in *D. purpurea* was 20.4% lower than in *Cucumis*; this reduction was exacerbated by continued stomatal opening after photosynthesis stabilized, leading to further inefficiency in water use. Limitation analysis further revealed contrasting dominant constraints: biochemical limitation accounted for 88.8% of total limitation in *Cucumis*, whereas stomatal limitation predominated in *D. purpurea* (64.3%). Together, these results highlight stomatal regulation as the primary bottleneck during photosynthetic induction in *D. purpurea*, leading to transient carbon losses and reduced water-use efficiency, providing a physiological basis for targeted cultivation strategies to improve both productivity and cardiotonic glycoside yield.

## 1. Introduction

Approximately 75% of the global population still relies on plants for traditional medicines, with *D. purpurea* serving as a prominent example [1]. As a biennial or perennial herb, *D. purpurea* has long been used to treat heart failure and cardiac edema [2,3]. Its principal bioactive compounds—cardiotonic glycosides—are structurally complex and challenging to synthesize chemically, making extraction from plants the primary production method. However, the cost remains high, reaching USD 3000 per kilogram [4], underscoring the urgent need to improve plant productivity and reduce production costs. In medicinal plants, photosynthesis not only determines biomass accumulation but also provides the carbon skeletons and energy required for secondary metabolite biosynthesis [5]. Enhancing photosynthetic performance in *D. purpurea* is therefore one of the most direct approaches to increasing yield and medicinal value.

Leaves are the principal sites of photosynthesis and secondary metabolite accumulation in *D. purpurea*. Photosynthesis encompasses photon capture by chloroplast light-harvesting complexes, energy conversion via electron transport and photophosphorylation, and CO_2_ fixation into triose phosphates that are subsequently converted into sucrose and starch [6]. Limitations to leaf photosynthesis are generally classified into CO_2_ diffusion limitations—stomatal and mesophyll conductance limitation—and biochemical limitations [7]. Stomatal traits, including size, density, and spatial distribution, influence stomatal conductance and thus regulate the trade-off between CO_2_ uptake and water loss [8]. In *D. purpurea*, stomatal density is responsive to environmental conditions: it decreases under full shade compared with semi-shaded conditions [9], increases in resistance following desiccation [10], and declines under low-vapor-pressure deficit (VPD) [11]. Such evidence suggests that manipulating stomatal behavior could be a viable strategy to improve photosynthetic performance. CO_2_ diffusion from the substomatal cavity to the chloroplast carboxylation sites is impeded by mesophyll resistance, the inverse of mesophyll conductance [12]. Notably, mesophyll conductance in *D. purpurea* appears insensitive to VPD and is relatively high compared with that of ferns [11,13].

Biochemical limitations are mainly associated with the activity of ribulose-1,5-bisphosphate carboxylase/oxygenase (Rubisco) and the capacity for ribulose-1,5-bisphosphate (RuBP) regeneration. Rubisco is inherently slow and requires CO_2_ for activation; the CO_2_ concentration is thus critical. Once carboxylation exceeds RuBP regeneration capacity, photosynthesis becomes limited by the latter [14,15]. Although little is known about biochemical capacity in *D. purpurea*, variations in Rubisco activity and cardiotonic glycoside content under different light spectra have been reported [16], yet no systematic study has examined its photosynthetic induction or the physiological basis of dynamic photosynthesis in this species, highlighting a critical knowledge gap directly relevant to its medicinal importance.

While most photosynthetic studies focus on steady-state conditions, plants in nature experience continuously fluctuating light, with irradiance being one of the most dynamic and influential environmental variables [17,18]. Photosynthetic induction—the transition from darkness or shade to light—is characterized by a lag before maximum CO_2_ assimilation is reached, leading to measurable carbon loss [19]. For example, Taylor’s study, based on a simple modeling approach, demonstrated that the presence of photosynthetic induction could lead to potential carbon assimilation losses of up to 21% in wheat [20]. Similarly, Wang et al., using ray-tracing techniques to capture canopy light dynamics combined with a complex metabolic model, reported that delayed photosynthetic responses during induction resulted in a C loss of 13% [21]. Therefore, accelerating photosynthetic induction responses represents an effective strategy to improve plant productivity by enhancing photosynthetic efficiency during this process. While significant progress has been made in elucidating the regulatory mechanisms of steady-state photosynthesis, the mechanisms underlying photosynthetic regulation during induction remain relatively less explored [22,23,24]. Specifically, during induction, stomatal conductance often responds more slowly than photosynthetic electron transport, while the gradual activation of Rubisco further constrains carbon fixation [25]. Stomatal adjustment is generally a slower process than the rise in *A*, thereby limiting the increase in assimilation during induction [26]. In some cases, net photosynthesis may already reach a steady state while stomatal conductance continues to increase, a phenomenon referred to by Lawson as stomatal overshoot [27]. This overshoot does not contribute to additional carbon gain but instead exacerbates water loss due to excessive stomatal opening, leading to a decline in WUE_i_. In contrast, the biochemical processes of electron transport and Rubisco activation stabilize more rapidly than stomatal responses [28,29].

Previous studies have also reported species-specific differences in the dominant limitations during photosynthetic induction. For instance, in rice and wheat, biochemical limitation is considered predominant, whereas in banana, stomatal limitation plays the major role [23,30,31,32]. However, in the case of *D. purpurea*, no study has yet investigated whether stomatal or biochemical processes represent the major bottleneck during photosynthetic induction. Addressing this gap is essential, not only for advancing fundamental understanding of its photosynthetic physiology but also for developing cultivation strategies that could enhance its medicinal value. Here, we compared *D. purpurea* with *Cucumis*, a high-yield herbaceous species from the same class that has undergone long-term domestication and intensive selection for productivity. Because of this breeding history, *Cucumis* is expected to exhibit inherently stronger photosynthetic induction capacity than *D. purpurea*. Our hypothesis is that the weaker induction response in *D. purpurea* reflects specific stomatal and/or biochemical constraints, which ultimately limit its productivity and cardiotonic glycoside accumulation. By explicitly linking photosynthetic induction dynamics with cardiotonic glycoside production, our study aims to identify the dominant limitations in *D. purpurea* and thereby provide a physiological basis for improving both its cultivation efficiency and its medicinal relevance.

## 2. Materials and Methods

### 2.1. Plant Materials and Growth Conditions

*D. purpurea* and *Cucumis* were grown outdoors under common garden conditions in 4.5 L pots filled with soil uniformly mixed with controlled-release NPK fertilizer (15-15-15, Osmocote, ICL Fertilizers Europe C.V., Heerlen, The Netherlands) at a rate of 2 g kg^−1^ dry soil. Plants were irrigated daily to field capacity to avoid drought stress, and pots were rotated weekly to minimize positional effects. After 30 days of growth, the plants were transferred to a greenhouse and acclimated for 3 days. During acclimation, the photosynthetic photon flux density (PPFD) was maintained above 1000 μmol m^−2^ s^−1^ by LED lamps. Daytime and nighttime temperatures in the greenhouse were maintained at 25 °C and 18 °C, respectively, with a relative humidity of 60%.

### 2.2. Measurement of Photosynthetic Induction

For the photosynthetic induction measurement, newly fully expanded leaves were selected, and the instrument used was the LI-6400 Photosynthesis System (LI-COR, Inc., Lincoln, NE, USA). For each species, six independent plants were cultivated as biological replicates. From these, three pots were randomly selected, and the most recently fully expanded leaf of each plant was used for gas exchange and chlorophyll fluorescence measurements (*n* = 3). Prior to induction, leaves were pre-illuminated at a low PPFD of 100 μmol m^−2^ s^−1^ for 20 min to achieve a steady physiological state. Previous studies have reported that rice genotypes can acclimate to low light within 2 min [33], suggesting that 20 min of pre-illumination provides ample time for stabilization across species. This procedure minimized potential variability due to dark-to-light transitions and ensured comparability among treatments. Subsequently, leaves were exposed to a high PPFD of 1000 μmol m^−2^ s^−1^ for 40 min. The reference CO_2_ concentration was set to 400 μmol mol^−1^, with a leaf temperature of 25 °C and a leaf-to-air VPD of 1 kPa. Gas exchange parameters—including *A*, *g*_s_, and intercellular CO_2_ concentration (*C_i_*)—were recorded every minute throughout both low- and high-light phases. WUE_i_ was calculated as the ratio of *A* to *g*_s_, and its mean value during high-light induction was defined as WUE_iave_. *A*_f_ and *g*_sf_ represent the maximum values of *A* and *g*_s_ observed under high light, respectively.

To assess biochemical limitation during photosynthetic induction, we adopted the assumption that biochemical constraints are primarily governed by the gradual activation of Rubisco. Previous studies have demonstrated that, following a transition from low to high irradiance, the kinetics of Rubisco carbonylation and the regeneration of its activator (RuBP) represent the dominant factors restricting carboxylation capacity. Accordingly, we considered Rubisco activation as the principal biochemical process underlying the delayed attainment of *V_cmax_*. The *V_cmax_* was calculated according to Deans using the following equation [34]:

(1) $$V_{\mathrm{cmax}}=\frac{\left(A\;+\;R_{d}){(C}_{i\;}+\;K_{m}\right)}{\left(C_{i}-\Gamma^{*}\right)}$$

where *R*_d_ is mitochondrial respiration in the light (set to 1 μmol m^−2^ s^−1^ following Luo et al.) [35], *K_m_* is the effective Michaelis–Menten constant for CO_2_ (541.9 μmol mol^−1^ at 25 °C), and Γ* is the CO_2_ compensation point in the absence of day respiration (40 μmol mol^−1^).

### 2.3. Quantification of Limitations During Photosynthetic Induction

Total deviation of *A* from its steady-state value (*dA*) was partitioned into stomatal (*dA*_s_) and biochemical (*dA*_b_) components as [34]:

(2) $$dA={dA}_{s}+{dA}_{b}=\frac{\partial A}{{\partial g}_{\mathrm{sc}}}dg_{\mathrm{sc}}+\frac{\partial A}{{\partial V}_{\mathrm{cmax}}}dV_{\mathrm{cmax}}.$$

Here, *g*_sc_ represents stomatal conductance to CO_2_ (*g*_s_/1.6). The changes *dg*_sc_ and *dV_cmax_* were calculated as the differences between observed values and steady-state values at the end of induction. The partial derivatives of *A* with respect to *g*_sc_ and *V_cmax_* were determined as:

(3) $$\frac{\partial A}{{\partial g}_{\mathrm{sc}}}=\frac{\frac{A}{g_{\mathrm{sc}}^{2}}\left((V_{\mathrm{cmax}}-R_{d}\right)-A)}{{(V}_{\mathrm{cmax}}-R_{d})(\frac{1}{g_{\mathrm{sc}}})+(C_{a}+K_{m})-2(\frac{1}{g_{\mathrm{sc}}})A}$$

(4) $$\frac{\partial A}{{\partial V}_{\mathrm{cmax}}}=\frac{\left(C_{a}-\Gamma^{*}\right)-A(\frac{1}{g_{\mathrm{sc}}})}{{(V}_{\mathrm{cmax}}-R_{d})(\frac{1}{g_{\mathrm{sc}}})+(C_{a}+K_{m})-2(\frac{1}{g_{\mathrm{sc}}})A}$$

The proportional contributions of stomatal (σ_s_) and biochemical (σ_b_) limitations to the total limitation were calculated as:

(5) $$\sigma_{s}\;=\;\frac{\int dA_{s}dt}{\int dA_{s}dt\;+\;\int dA_{b}dt}$$

(6) $$\sigma_{b}=1-\sigma_{s}$$

### 2.4. Photosynthetic Induction Kinetics, Carbon Gain, and Potential Carbon Loss

The kinetics of photosynthetic induction parameters were fitted using an exponential model [36]:

(7) $$X\left(t\right)=X_{f}+(X_{i}-X_{f})e^{\frac{-t}{\tau }}$$

where *X*_i_ and *X*_f_ are the initial and final values of a given parameter (e.g., *A* or *V_cmax_*) during induction, and τ is the time constant. The time to reach 90% of the final value was calculated as:

(8) $$t_{90}=-\tau *\ln\left(0.1\right)$$

The cumulative carbon gain during induction was calculated as:

(9) $$C\;gain=\int_{0}^{t}A\left(t\right)dt$$

The C loss ratio representing the percentage of potential carbon assimilation lost due to delayed induction which was calculated as:

(10) $$C\;loss\;ratio=\frac{A_{f}*t-C\;gain}{A_{f}}*100$$

### 2.5. Statistical Analysis

Prior to statistical analyses, data were tested for normality using the Shapiro–Wilk test and for homogeneity of variance using Levene’s test. As both assumptions were satisfied, differences in physiological parameters between *Digitalis* and *Cucumis* were analyzed using independent-samples *t*-tests, which are statistically equivalent to one-way ANOVA when only two groups are compared. Since only two species were involved, no post hoc tests were required. Comparisons of stomatal and biochemical limitations within each species were also performed using Student’s *t*-test. All figures and regression analyses were prepared in OriginPro 2025. Data are presented as means ± standard deviation.

## 3. Results

### 3.1. Variations in Photosynthetic Parameters During Induction

**Figure 1:**
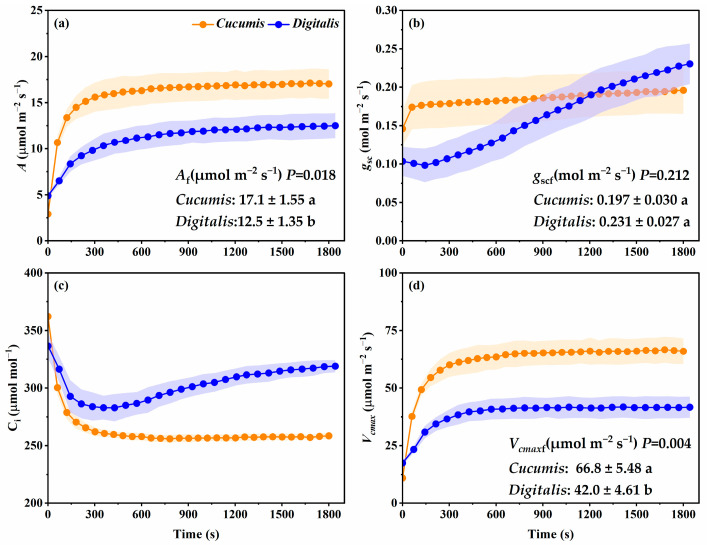
Dynamic changes in photosynthetic parameters of *Cucumis* and *Digitalis* leaves, during photosynthetic induction under a photosynthetic photon flux density of 1000 μmol m^−2^ s^−1^. (**a**) *A*, net photosynthetic rate; (**b**) *g*_sc_, stomatal conductance to CO_2_; (**c**) *C*_i_, intercellular CO_2_ concentration; (**d**) *V*_cmax_, the maximum carboxylation rate. (**a**) *A*_f_, (**b**) *g*_scf_, and (**d**) *V_cmax_*_f_ were the steady-state *A*, *g*_sc_, and *V_cmax_* of the last 1 min of light induction period (1000 μmol m^−2^ s^−1^). Statistical significance was determined by Student’s *t*-test. Exact *p*-values are shown in the figure, and different letters indicate significant differences at *p* < 0.05. Data are means ± SD (*n* = 3).

Figure 1 shows the dynamic changes in photosynthesis in *Cucumis* and *D. purpurea* under a PPFD of 1000 μmol m^−2^ s^−1^. In both species, *A* increased progressively with induction time and eventually reached distinct steady-state values (Figure 1a). Throughout the induction period, *D. purpurea* maintained significantly lower *A* compared with *Cucumis*. This persistent gap suggests that the medicinal species operates with an intrinsically lower photosynthetic capacity, which could constrain biomass accumulation and metabolite yield under field conditions. The *g*_sc_ in *Cucumis* increased rapidly after the onset of induction and then stabilized. In contrast, *D. purpurea* showed a transient decline in *g*_sc_ at the beginning of induction, followed by a gradual increase, reflecting a markedly slower stomatal response (Figure 1b). Moreover, even after photosynthesis had stabilized, *g*_sc_ in *D. purpurea* continued to rise, exhibiting the characteristic stomatal overshoot. Such delayed and excessive stomatal adjustment not only reduces carbon gain but also increases water loss, highlighting a critical inefficiency in gas-exchange regulation. Correspondingly, *C*_i_ in *D. purpurea* increased after a brief initial decline, indicating reduced mesophyll CO_2_ utilization, whereas in *Cucumis*, *C*_i_ rapidly decreased and stabilized at 258.5 μmol mol^−1^, consistent with more efficient CO_2_ assimilation (Figure 1c). These trends were further supported by the *V_cmax_* (Figure 1d): *Cucumis* rapidly achieved and sustained a high *V_cmax_* throughout induction, while *D. purpurea* maintained substantially lower values. Taken together, these differences underscore that both stomatal and biochemical processes limit the efficiency of photosynthetic induction in *D. purpurea*, with direct consequences for its growth performance.

### 3.2. Photosynthetic Traits at Steady State

**Figure 2:**
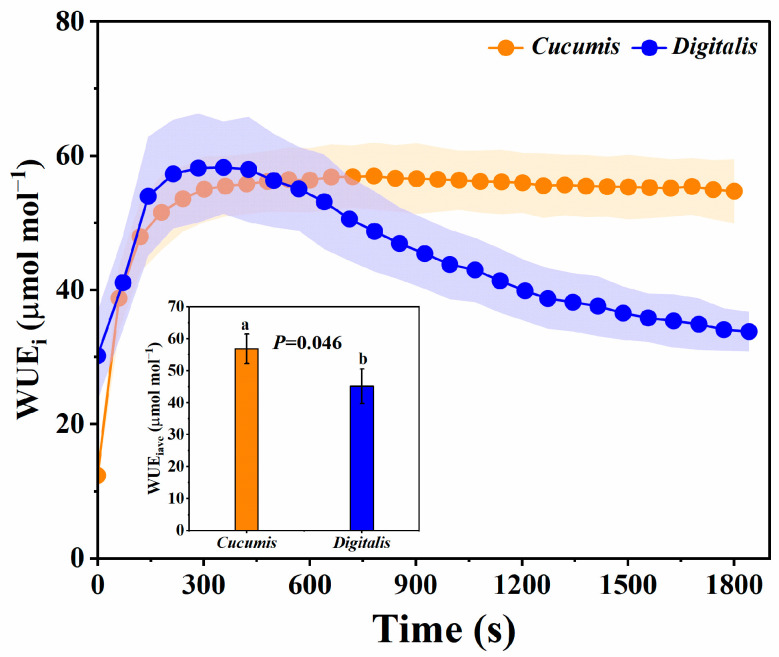
Dynamic changes in water use efficiency of *Cucumis* and *Digitalis* during photosynthetic induction. WUE_iave_, average intrinsic water-use efficiency. Statistical significance was determined by Student’s *t*-test. Exact *p*-values are shown in the figure, and different letters indicate significant differences at *p* < 0.05. Data are means ± SD (*n* = 3).

Analysis of steady-state photosynthetic parameters revealed that *D. purpurea* consistently underperformed relative to *Cucumis*, indicating considerable potential for improvement (Figure 1). The *A*_f_ of *D. purpurea* was 12.5 μmol m^−2^ s^−1^, which was significantly lower than that of *Cucumis* (17.1 μmol m^−2^ s^−1^) (Figure 1) and only accounted for 73.1% of *Cucumis*’s *A*_f_.The *g*_scf_ in *D. purpurea* reached 0.230 mol m^−2^ s^−1^, slightly higher than *Cucumis*’s 0.197 mol m^−2^ s^−1^, though the difference was not statistically significant; notably, *D. purpurea* required 1200 s to reach a *g*_sc_ level comparable to *Cucumis* (Figure 1b and Figure 2). Similarly, *V_cmax_*_f_ in *D. purpurea* was markedly lower than in *Cucumis*—42.0 μmol m^−2^ s^−1^ versus 66.8 μmol m^−2^ s^−1^—indicating reduced carboxylation capacity, reaching only 62.9% of that of *Cucumis* (Figure 1d).

### 3.3. Dynamic Changes in Water Use Efficiency

**Figure 3:**
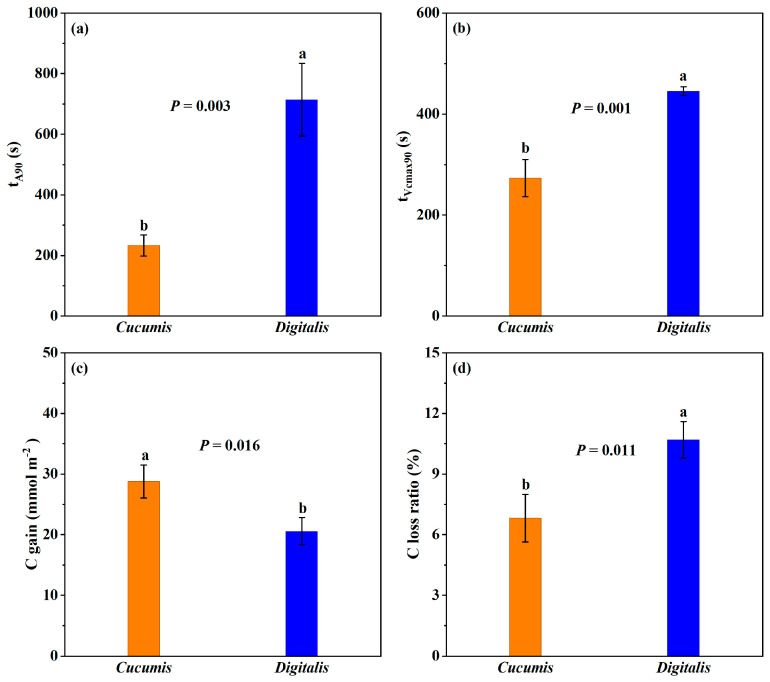
Dynamic photosynthetic parameters of *Cucumis* and *Digitalis* during photosynthetic induction. (**a**) t_A90_, (**b**) t_Vcmax90_ were the time to reach 90% induction state of net photosynthetic rate (*A*) and maximum carboxylation rate (*V_cmax_*); (**c**) cumulative carbon gain during the 40 min induction; (**d**) carbon loss ratio. Statistical significance was determined, followed by Student’s *t*-test. Exact *p*-values are shown in the figure, and different letters indicate significant differences at *p* < 0.05. Data are means ± SD (*n* = 3).

The dynamics of WUE_i_ during induction are shown in Figure 3. In *Cucumis*, WUE_i_ increased rapidly, stabilizing at a high level (>50) after ~300 s. In *D. purpurea*, WUE_i_ also peaked around 300 s but then declined steadily, eventually approaching its pre-induction level (~30 μmol mol^−1^). This pattern indicates unstable water use under high-light induction in *D. purpurea*. WUE_iave_ was 20.4% lower in *D. purpurea* than in *Cucumis*, confirming its comparatively lower water use efficiency and indicating that *D. purpurea* exhibits potential for improving water-use efficiency during photosynthetic induction under dynamic light fluctuations.

### 3.4. Response Speed to Reach 90% Induction State

The time required to reach 90% of the maximum induction level differed sharply between species (Figure 3). For *A*, *D. purpurea* required 714 s—approximately three times longer than *Cucumis*’s 233 s (Figure 3a). Similarly, the time to reach 90% of maximum *V_cmax_* was ~446 s in *D. purpurea*, 1.6 times that of *Cucumis* (Figure 3b). Cumulative carbon gain (C gain) over the 40 min induction period was 28.8 mmol m^−2^ in *Cucumis* but only 20.6 mmol m^−2^ in *D. purpurea* (Figure 3c). The calculated C loss ratio was 10.7% for *D. purpurea*, significantly higher than *Cucumis*’s 6.8%. Overall, during photosynthetic induction, *D. purpurea* not only exhibits lower cumulative carbon assimilation compared with *Cucumis*, but also suffers from higher C loss. This dual disadvantage highlights the need for simultaneous improvement in both assimilation efficiency and the reduction in induction-related carbon losses.

### 3.5. Dynamic and Time-Integrated Photosynthetic Limitations

**Figure 4:**
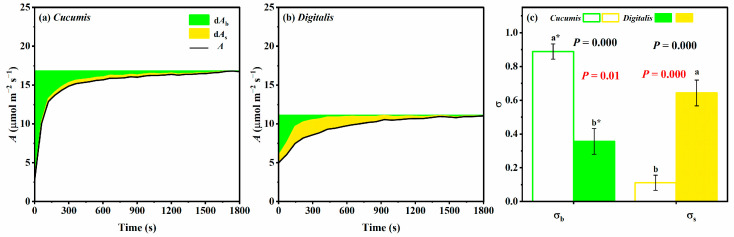
Dynamic variations in the photosynthetic limitation and the time-integrated limitations of *Cucumis* and *Digitalis* during photosynthetic induction. (**a**) d*A*_b_ and d*A*_s_ were the biochemical limitation and stomatal limitations of *Cucumis* during photosynthetic induction, respectively; (**b**) d*A*_b_ and d*A*_s_ were the biochemical limitation and stomatal limitations of *Digitalis* during photosynthetic induction, respectively. (**c**) σ_b_: The time-integrated biochemical limitations in response to photosynthetic induction; σ_s_: the time-integrated stomatal limitation in response to photosynthetic induction. Statistical significance was determined by Student’s *t*-test. Exact *p*-values are shown in the figure: Black font denotes comparisons between *Cucumis* and *D. purpurea* and red font denotes comparisons between σ_b_ and σ_s_ within each species. Asterisks indicate significant differences between stomatal and biochemical limitations within the same species (*p* < 0.05). Different lowercase letters denote significant differences in stomatal or biochemical limitation between *Cucumis* and *Digitalis*. Data are presented as means ± SD (*n* = 3).

Limitation analysis revealed contrasting patterns between species during induction (Figure 4). In *Cucumis*, biochemical limitation (d*A*_b_) dominated throughout induction, peaking at the start and declining rapidly within 300 s to a relatively low level; stomatal limitation (d*A*_s_) remained consistently weaker (Figure 4a). In *D. purpurea*, d*A*_b_ was initially the main limiting factor but shifted toward stomatal limitation after ~100 s, then reverted to biochemical limitation dominance in the later stages. Notably, d*A*_s_ was already substantial at induction onset—unlike in *Cucumis* (Figure 4b). Time-integrated analysis showed that stomatal limitation accounted for 64.3% of total limitation in *D. purpurea*, significantly exceeding biochemical limitation. In *Cucumis*, biochemical limitation contributed 88.8% of the total (Figure 4c). These differences highlight stomatal traits as the primary targets for improving photosynthetic induction in *D. purpurea*.

## 4. Discussion

### 4.1. Slower Induction Rates in D. purpurea Compared with Those of Cucumis Result in Greater Carbon Loss

Even under clear and windless conditions, leaves may experience hundreds of short-lived sunflecks (10–120 s) throughout the day. It has been well established that such transient light fluctuations are major contributors to understory photosynthesis, accounting for 10–80% of the total PPFD received by plants [17,19,37,38]. Consequently, slower photosynthetic induction rates tend to cause greater carbon losses. Previous studies have reported that the induction process can account for carbon losses of up to 20% in soybean (*Glycine max* L. Merr.) and wheat (*Triticum aestivum* L.) under simulated natural light fluctuations in the field [20,39].

Consistent with these reports, *D. purpurea* exhibited a significantly slower induction response (Figure 3a,b) than *Cucumis*, with longer times required to reach 90% of both the steady-state *A* and the *V_cmax_*. This delay led to a C loss ratio of 11.5% during the induction period (Figure 3d). In addition to the higher proportional carbon loss, *D. purpurea* also showed a lower total carbon gain (Figure 3c), which was closely related to its reduced CO_2_ carboxylation rate (Figure 1). CO_2_ reaching the chloroplast carboxylation sites enters the Calvin–Benson–Bassham cycle to produce carbohydrates, a process regulated by biochemical factors. Importantly, the rate is determined not by the total Rubisco content but by the proportion of Rubisco in the activated state [40]. The activated Rubisco in *D. purpurea* determines the maximum amount of CO_2_ that can be carboxylated at any given time (i.e., *V_cmaxf_*, Figure 1), and thus sets the upper limit of its *A* (Figure 1). This biochemical bottleneck reduces assimilation efficiency, ultimately affecting both biomass accumulation and metabolite production.

### 4.2. Stomatal Limitation as the Primary Constraint on Photosynthetic Induction in D. purpurea

During photosynthetic induction, the balance between stomatal limitation and biochemical limitation plays a crucial regulatory role in determining photosynthetic performance. Previous studies have suggested that when *g*_s_ cannot meet the CO_2_ demand of photosynthesis, stomatal limitation becomes the dominant factor restricting photosynthetic capacity [41]. Conversely, when CO_2_ supply is sufficient but the biochemical machinery cannot support further assimilation, biochemical limitation becomes predominant [42]. The dominant factor therefore varies depending on species and environmental conditions. It is worth noting that many studies have applied limitation analysis developed under steady-state light conditions, which provides only relative limitations at individual time points [23,31]. However, during photosynthetic induction, the amount of CO_2_ assimilation lost at each time point is not uniform, making such approaches insufficient to capture the true constraints of the process. To overcome this, a time-integrated limitation analysis is required, which accounts for the cumulative impact of different factors over the entire induction period. Although this approach was first introduced by Deans in 2019, its application remains rare [34]. In the present study, we employed time-integrated limitation analysis to identify the major factors constraining photosynthetic induction in *Cucumis* and *D. purpurea*. Consistent with this pattern, our results show that *D. purpurea* was primarily limited by stomatal factors, while *Cucumis* was mainly limited by biochemical processes (Figure 4c). In *Cucumis*, a rapid stomatal response (Figure 1b) was well coupled with *A* (Figure 1a), ensuring a stable CO_2_ supply during induction; thus, biochemical capacity was the main constraint, and enhancing biochemical processes could further boost photosynthetic performance. In contrast, *D. purpurea* exhibited a slower stomatal opening response (Figure 1b), which was poorly synchronized with changes in *A* (Figure 1a), leading to a pronounced stomatal limitation. This asynchrony between *A* and *g*_s_ also contributed to the progressive decline in instantaneous WUE_i_ (Figure 2), suggesting a potential risk of excessive water loss under high-light induction, as the rate of transpiration water loss through stomata increased more rapidly than CO_2_ assimilation [43]. Previous work has shown that faster stomatal kinetics during induction not only enhance WUE_i_ but also enable greater biomass accumulation, ultimately benefiting metabolite yield [44]. This has direct relevance to *D. purpurea*, where improved stomatal responsiveness could enhance both carbon gain and the production of valuable secondary metabolites such as cardenolides. We acknowledge that mesophyll conductance (*g*_m_) also plays a role during photosynthetic induction. Accordingly, the biochemical limitation derived from our time-integrated limitation analysis represents an apparent biochemical limitation that implicitly includes the effect of *g*_m_. However, in *D. purpurea*, stomatal limitation accounted for as much as 64.3% of the total limitation, indicating that further quantification of *g*_m_ would not alter the conclusion that stomatal constraints predominate. This provides an important implication for the community: in laboratories lacking fluorescence-equipped leaf chambers, photosynthetic induction measurements based solely on gas exchange may still be sufficient, as quantifying *g*_m_ becomes unnecessary when stomatal limitation is clearly dominant.

It is also important to note that the relative contributions of stomatal and biochemical limitations are not static but fluctuate with environmental and physiological conditions. For instance, Tang et al. reported that elevated CO_2_ can shift the dominant limitation from stomatal to biochemical in *Populus tomentosa* and *Eucalyptus robusta* [45]. Similarly, Tomimatsu et al. observed that at higher CO_2_ concentrations, biochemical limitations—particularly electron transport and RuBP regeneration—become increasingly important [46]. In *D. purpurea*, we also observed temporal shifts in the dominant limitation during induction (Figure 4b): biochemical limitation was prominent in both early and late induction phases, especially in the latter stage when *g*_s_ increased sufficiently to ensure adequate CO_2_ supply (Figure 1c and Figure 2), at which point the relatively low *V_cmax_* became the major constraint. Therefore, although stomatal limitation is the primary constraint during photosynthetic induction in *D. purpurea*, biochemical limitations should not be overlooked. Under relatively stable light environments, biochemical capacity may become the dominant factor limiting photosynthetic performance. To more accurately simulate the dynamic light environment found in nature, future studies should establish controlled indoor experiments that manipulate the duration of lightflecks to investigate their impact on photosynthetic induction and biomass.

### 4.3. Cultivation Recommendations for D. purpurea

The photosynthetic capacity of *D. purpurea* leaves is closely linked to the synthesis of cardenolides, and leaf biomass directly influences their yield. Based on our results, for indoor greenhouse cultivation, maintaining a stable light source is critical for avoiding carbon loss during photosynthetic induction. In terms of LED light spectrum composition, moderately increasing the proportion of blue light may promote cardenolide synthesis, as blue light photoreceptors—together with protochlorophyllide holochrome or phytochrome—are involved in regulating cardenolide biosynthesis and accumulation [47]. Ensuring sufficient CO_2_ supply and maintaining optimal humidity to promote stomatal opening are effective strategies for reducing stomatal limitation. Likewise, controlling day–night temperatures to sustain the activity of photosynthetic enzymes is essential for minimizing biochemical limitations under steady-state photosynthesis. For outdoor cultivation, *D. purpurea* should be planted in open areas with appropriate planting density to minimize shading, thereby maintaining a stable light environment. Supplemental irrigation methods, such as sprinkler systems, can enhance Rubisco activity [48]. Additionally, targeted breeding and genetic engineering approaches offer promising avenues for improving *D. purpurea* photosynthetic traits, enabling enhanced carbon gain and metabolite production.

## 5. Conclusions

The results demonstrate that, relative to *Cucumis*, *Digitalis* exhibits lower *A* and carbon assimilation during induction, together with reduced water-use efficiency. *Digitalis* also requires a longer period to attain steady-state photosynthesis and incurs greater potential carbon losses during induction. Limitation analysis further revealed contrasting dominant factors between the two species: biochemical limitation prevailed in *Cucumis*, whereas stomatal limitation was predominant in *Digitalis*. This finding not only provides a valuable reference for optimizing *Digitalis* cultivation—suggesting that enhancing stomatal regulation and biochemical capacity could improve leaf biomass accumulation and cardiac glycoside production—but also carries methodological implications. Specifically, in studies of photosynthetic induction, gas-exchange measurements alone may be sufficient. When stomatal limitation is clearly identified as the dominant factor, further quantification of mesophyll conductance becomes unnecessary, offering a practical approach particularly for laboratories without access to fluorescence-equipped systems.

## Data Availability

Data are contained within the article.
